# (*E*)-*N*′-(3-Hy­droxy-4-meth­oxy­benzyl­idene)-4-meth­oxy­benzohydrazide

**DOI:** 10.1107/S1600536811048240

**Published:** 2011-11-19

**Authors:** Hoong-Kun Fun, Premrudee Promdet, Suchada Chantrapromma, Jirapa Horkaew, Chatchanok Karalai

**Affiliations:** a X-ray Crystallography Unit, School of Physics, Universiti Sains Malaysia, 11800 USM, Penang, Malaysia; b Crystal Materials Research Unit, Department of Chemistry, Faculty of Science, Prince of Songkla University, Hat-Yai, Songkhla 90112, Thailand

## Abstract

The title mol­ecule, a benzohydrazide derivative, C_16_H_16_N_2_O_4_, is twisted with a dihedral angle of 69.97 (5)° between the two benzene rings. An intra­molecular O—H⋯O hydrogen bond generates an *S*(5) ring motif. In the crystal, mol­ecules are linked by N—H⋯O and weak C—H⋯O hydrogen bonds into a chain along the *c* axis. C—H⋯π inter­actions are also present.

## Related literature

For bond-length data, see: Allen *et al.* (1987 ▶). For details of hydrogen-bond motifs, see: Bernstein *et al.* (1995 ▶). For related structures, see: Fun *et al.* (2011 ▶); Horkaew *et al.* (2011 ▶); Promdet *et al.* (2011 ▶). For background and applications of benzohydrazide derivatives, see: Bedia *et al.* (2006 ▶); Loncle *et al.* (2004 ▶); Melnyk *et al.* (2006 ▶); Raj *et al.* (2007 ▶). For the stability of the temperature controller used in the data collection, see: Cosier & Glazer (1986 ▶).
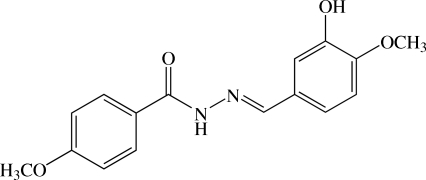


## Experimental

### 

#### Crystal data

C_16_H_16_N_2_O_4_
*M* *_r_* = 300.31
Monoclinic, 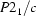

*a* = 12.1323 (19) Å
*b* = 12.9727 (15) Å
*c* = 9.6714 (12) Å
β = 113.213 (2)°
*V* = 1398.9 (3) Å^3^
*Z* = 4
Mo *K*α radiation
μ = 0.10 mm^−1^
*T* = 100 K
0.58 × 0.30 × 0.07 mm

#### Data collection

Bruker APEX DUO CCD area-detector diffractometer
Absorption correction: multi-scan (*SADABS*; Bruker, 2009 ▶) *T*_min_ = 0.942, *T*_max_ = 0.993
14521 measured reflections
3704 independent reflections
3260 reflections with *I* > 2σ(*I*)
*R*_int_ = 0.021

#### Refinement

*R*[*F* ^2^ > 2σ(*F* ^2^)] = 0.037
*wR*(*F* ^2^) = 0.109
*S* = 1.03
3704 reflections
208 parameters
H atoms treated by a mixture of independent and constrained refinement
Δρ_max_ = 0.39 e Å^−3^
Δρ_min_ = −0.23 e Å^−3^

### 

Data collection: *APEX2* (Bruker, 2009 ▶); cell refinement: *SAINT* (Bruker, 2009 ▶); data reduction: *SAINT*; program(s) used to solve structure: *SHELXTL* (Sheldrick, 2008 ▶); program(s) used to refine structure: *SHELXTL*; molecular graphics: *SHELXTL*; software used to prepare material for publication: *SHELXTL* and *PLATON* (Spek, 2009 ▶).

## Supplementary Material

Crystal structure: contains datablock(s) global, I. DOI: 10.1107/S1600536811048240/is5009sup1.cif

Structure factors: contains datablock(s) I. DOI: 10.1107/S1600536811048240/is5009Isup2.hkl

Supplementary material file. DOI: 10.1107/S1600536811048240/is5009Isup3.cml

Additional supplementary materials:  crystallographic information; 3D view; checkCIF report

## Figures and Tables

**Table 1:** Hydrogen-bond geometry (Å, °) *Cg*1 is the centroid of the C1–C6 ring.

*D*—H⋯*A*	*D*—H	H⋯*A*	*D*⋯*A*	*D*—H⋯*A*
O3—H1*O*3⋯O4	0.87 (2)	2.18 (2)	2.6692 (13)	115.9 (18)
N1—H1*N*1⋯O1^i^	0.887 (18)	1.992 (18)	2.8698 (13)	170.0 (16)
C8—H8*A*⋯O1^i^	0.93	2.47	3.2780 (15)	145
C15—H15*C*⋯*Cg*1^ii^	0.96	2.72	3.5664 (15)	148
C16—H16*B*⋯*Cg*1^iii^	0.96	2.76	3.4366 (16)	128

Symmetry codes: (i) 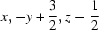; (ii) 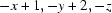; (iii) 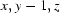.

**Fig. 1.**
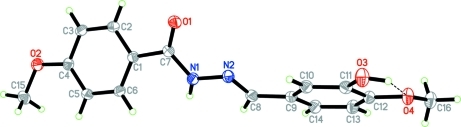
The molecular structure of the title compound, showing 60% probability displacement ellipsoids and the atom-numbering scheme. Hydrogen bond was drawn as a dash line.

**Fig. 2.**
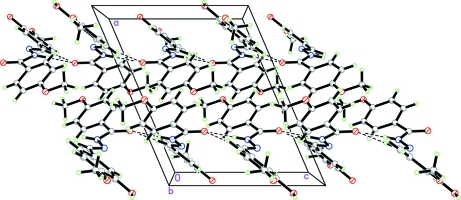
A crystal packing diagram of the title compound viewed along the b axis, showing chains running along the c axis. Hydrogen bonds were drawn as dashed lines.

## supplementary crystallographic information

### Comment

Benzohydrazide derivatives have been reported to possess various biological properties, such as antibacterial and antifungal (Loncle *et al.*, 2004), antitubercular (Bedia *et al.*, 2006), antimalarial (Melnyk *et al.*, 2006) and antiproliferative (Raj *et al.*, 2007) activities. We have previously reported some crystal structures of this type of compounds (Fun *et al.*, 2011; Horkaew *et al.*, 2011; Promdet *et al.*, 2011). The title compound (I) was synthesized in order to study the effect of functional groups to their bioactivities comparing to the closely related structures. (I) was screened for antibacterial and antioxidant activities. Our results show that (I) exhibits moderate antibacterial activity whereas it is inactive for antioxidant activity. The three dimensional structure of (I) was studied in order to gain more details to explain the effect of structure on its bioactivity.

The molecule of the title benzohydrazide derivative (Fig. 1), C_16_H_16_N_2_O_4_, exists in a *trans*-configuration with respect to the C8═N2 bond [1.2808 (14) Å] and the torsion angle of N1–N2–C8–C9 is 179.20 (9)°. The molecule is twisted as indicated by the dihedral angle between the two benzene rings being 69.97 (5)°. The middle bridge fragment (O1/C7/N1/N2/C8) is nearly planar with a torsion angle N2–N1–C7–O1 = -0.80 (16)°. The mean plane through this bridge makes dihedral angles of 27.88 (7) and 43.44 (7)° with the C1–C6 and C9–C14 benzene rings, respectively. The methoxy group of 4-methoxyphenyl (at atom C4) is co-planar with its bound benzene ring [torsion angle C15–O2–C4–C5 = -0.76 (16)° and *r.m.s* 0.0131 (1) Å for the seven non H atoms], whereas the methoxy group of the 3-hydroxy-4-methoxyphenyl (at atom C12) is slight deviated with a torsion angle C16–O4–C12–C13 = 10.02 (15)°. An intramolecular O3—H1O3···O4 hydrogen bond generates an S(5) ring motif (Bernstein *et al.*, 1995). Bond distances are of normal values (Allen *et al.*, 1987) and are comparable with the related structures (Fun *et al.*, 2011; Horkaew *et al.*, 2011; Promdet *et al.*, 2011).

In the crystal packing (Fig. 2), the molecules are linked by N—H···O hydrogen bonds and weak C—H···O interactions (Table 1) into chains along the *c* axis. These chains are arranged in a face-to-face manner. The crystal is stabilized by N—H···O hydrogen bonds, weak C—H···O and C—H···π interactions (Table 1).

### Experimental

The title compound (I) was prepared by dissolving 4-methoxybenzohydrazide (2 mmol, 0.33 g) in ethanol (10 ml). The solution of 3-hydroxy-4-methoxybenzaldehyde (2 mmol, 0.30 g) in ethanol (10 ml) was then added slowly to the reaction. The mixture was refluxed for around 3 hr. The solution was then cooled to room temperature. Colorless plate-shaped single crystals of the title compound suitable for *X*-ray structure determination were recrystallized from methanol by slow evaporation of the solvent at room temperature after several days (m.p. 491-492 K).

### Refinement

Amide and hydroxy H atoms were located in difference maps and refined isotropically [N—H = 0.887 (18) Å and O—H = 0.87 (2) Å]. The remaining H atoms were positioned geometrically and allowed to ride on their parent atoms, with C—H = 0.93 Å for aromatic and CH and 0.96 Å for CH_3_ atoms. The *U*_iso_ values were constrained to be 1.5*U*_eq_ of the carrier atom for methyl H atoms and 1.2*U*_eq_ for the remaining H atoms. A rotating group model was used for the methyl groups.

### Crystal data

**Table:**

C_16_H_16_N_2_O_4_	*F*(000) = 632
*M**_r_* = 300.31	*D*_x_ = 1.426 Mg m^−^^3^
Monoclinic, *P*2_1_/*c*	Melting point = 491–492 K
Hall symbol: -P 2ybc	Mo *K*α radiation, λ = 0.71073 Å
*a* = 12.1323 (19) Å	Cell parameters from 3704 reflections
*b* = 12.9727 (15) Å	θ = 1.8–29.0°
*c* = 9.6714 (12) Å	µ = 0.10 mm^−^^1^
β = 113.213 (2)°	*T* = 100 K
*V* = 1398.9 (3) Å^3^	Plate, colorless
*Z* = 4	0.58 × 0.30 × 0.07 mm

### Data collection

**Table:**

Bruker APEX DUO CCD area-detector diffractometer	3704 independent reflections
Radiation source: sealed tube	3260 reflections with *I* > 2σ(*I*)
graphite	*R*_int_ = 0.021
φ and ω scans	θ_max_ = 29.0°, θ_min_ = 1.8°
Absorption correction: multi-scan (*SADABS*; Bruker, 2009)	*h* = −15→16
*T*_min_ = 0.942, *T*_max_ = 0.993	*k* = −17→16
14521 measured reflections	*l* = −13→13

### Refinement

**Table:**

Refinement on *F*^2^	Primary atom site location: structure-invariant direct methods
Least-squares matrix: full	Secondary atom site location: difference Fourier map
*R*[*F*^2^ > 2σ(*F*^2^)] = 0.037	Hydrogen site location: inferred from neighbouring sites
*wR*(*F*^2^) = 0.109	H atoms treated by a mixture of independent and constrained refinement
*S* = 1.03	*w* = 1/[σ^2^(*F*_o_^2^) + (0.058*P*)^2^ + 0.5751*P*] where *P* = (*F*_o_^2^ + 2*F*_c_^2^)/3
3704 reflections	(Δ/σ)_max_ = 0.001
208 parameters	Δρ_max_ = 0.39 e Å^−^^3^
0 restraints	Δρ_min_ = −0.23 e Å^−^^3^

### Special details

**Table:**

Experimental. The crystal was placed in the cold stream of an Oxford Cryosystems Cobra open-flow nitrogen cryostat (Cosier & Glazer, 1986) operating at 100.0 (1) K.
Geometry. All esds (except the esd in the dihedral angle between two l.s. planes) are estimated using the full covariance matrix. The cell esds are taken into account individually in the estimation of esds in distances, angles and torsion angles; correlations between esds in cell parameters are only used when they are defined by crystal symmetry. An approximate (isotropic) treatment of cell esds is used for estimating esds involving l.s. planes.
Refinement. Refinement of F^2^ against ALL reflections. The weighted R-factor wR and goodness of fit S are based on F^2^, conventional R-factors R are based on F, with F set to zero for negative F^2^. The threshold expression of F^2^ > 2sigma(F^2^) is used only for calculating R-factors(gt) etc. and is not relevant to the choice of reflections for refinement. R-factors based on F^2^ are statistically about twice as large as those based on F, and R- factors based on ALL data will be even larger.

### Fractional atomic coordinates and isotropic or equivalent isotropic displacement parameters (Å^2^)

**Table:**

	*x*	*y*	*z*	*U*_iso_*/*U*_eq_	
O1	0.29470 (7)	0.79595 (6)	0.37018 (8)	0.01765 (17)	
O2	0.47609 (8)	1.16425 (6)	0.09037 (9)	0.02033 (18)	
O3	−0.02649 (8)	0.33066 (7)	0.26553 (10)	0.02208 (19)	
H1O3	−0.0427 (18)	0.2655 (17)	0.262 (2)	0.049 (6)*	
O4	0.05699 (8)	0.16672 (6)	0.17060 (10)	0.02052 (18)	
N1	0.24563 (9)	0.72966 (7)	0.13517 (10)	0.01662 (19)	
H1N1	0.2597 (15)	0.7299 (13)	0.0519 (19)	0.027 (4)*	
N2	0.20136 (8)	0.63908 (7)	0.16926 (10)	0.01754 (19)	
C1	0.33802 (9)	0.89609 (8)	0.19231 (11)	0.0143 (2)	
C2	0.42433 (10)	0.95553 (8)	0.30308 (11)	0.0169 (2)	
H2A	0.4511	0.9355	0.4033	0.020*	
C3	0.47027 (10)	1.04352 (8)	0.26570 (11)	0.0175 (2)	
H3A	0.5285	1.0818	0.3403	0.021*	
C4	0.42906 (9)	1.07495 (8)	0.11550 (12)	0.0158 (2)	
C5	0.34465 (9)	1.01566 (8)	0.00388 (11)	0.0162 (2)	
H5A	0.3184	1.0355	−0.0964	0.019*	
C6	0.29974 (9)	0.92674 (8)	0.04258 (11)	0.0155 (2)	
H6A	0.2434	0.8872	−0.0323	0.019*	
C7	0.29096 (9)	0.80337 (8)	0.24151 (11)	0.0143 (2)	
C8	0.20399 (10)	0.56293 (9)	0.08631 (12)	0.0174 (2)	
H8A	0.2331	0.5735	0.0117	0.021*	
C9	0.16289 (9)	0.46024 (8)	0.10510 (11)	0.0167 (2)	
C10	0.08516 (9)	0.44367 (8)	0.17847 (11)	0.0170 (2)	
H10A	0.0565	0.4994	0.2150	0.020*	
C11	0.05118 (9)	0.34488 (8)	0.19644 (12)	0.0168 (2)	
C12	0.09594 (9)	0.26028 (8)	0.14405 (12)	0.0167 (2)	
C13	0.17260 (10)	0.27633 (9)	0.07125 (12)	0.0192 (2)	
H13A	0.2022	0.2205	0.0362	0.023*	
C14	0.20503 (10)	0.37620 (9)	0.05082 (12)	0.0192 (2)	
H14A	0.2554	0.3869	0.0004	0.023*	
C15	0.43662 (11)	1.19826 (9)	−0.06160 (13)	0.0221 (2)	
H15A	0.4779	1.2606	−0.0655	0.033*	
H15B	0.3518	1.2110	−0.1008	0.033*	
H15C	0.4535	1.1461	−0.1208	0.033*	
C16	0.11532 (11)	0.07892 (9)	0.14054 (14)	0.0228 (2)	
H16A	0.0817	0.0171	0.1624	0.034*	
H16B	0.1995	0.0817	0.2025	0.034*	
H16C	0.1039	0.0790	0.0365	0.034*	

### Atomic displacement parameters (Å^2^)

**Table:**

	*U*^11^	*U*^22^	*U*^33^	*U*^12^	*U*^13^	*U*^23^
O1	0.0231 (4)	0.0179 (4)	0.0150 (3)	0.0017 (3)	0.0108 (3)	0.0010 (3)
O2	0.0243 (4)	0.0178 (4)	0.0183 (4)	−0.0057 (3)	0.0077 (3)	0.0011 (3)
O3	0.0220 (4)	0.0190 (4)	0.0318 (4)	−0.0008 (3)	0.0175 (3)	0.0007 (3)
O4	0.0227 (4)	0.0151 (4)	0.0284 (4)	−0.0019 (3)	0.0150 (3)	0.0001 (3)
N1	0.0227 (4)	0.0154 (4)	0.0144 (4)	−0.0033 (3)	0.0100 (3)	−0.0004 (3)
N2	0.0195 (4)	0.0164 (4)	0.0166 (4)	−0.0041 (3)	0.0070 (3)	0.0013 (3)
C1	0.0175 (5)	0.0137 (4)	0.0136 (4)	0.0006 (4)	0.0082 (4)	−0.0006 (3)
C2	0.0209 (5)	0.0175 (5)	0.0125 (4)	0.0000 (4)	0.0066 (4)	−0.0009 (4)
C3	0.0197 (5)	0.0172 (5)	0.0147 (4)	−0.0028 (4)	0.0058 (4)	−0.0029 (4)
C4	0.0172 (5)	0.0145 (5)	0.0176 (5)	−0.0006 (4)	0.0088 (4)	0.0002 (4)
C5	0.0183 (5)	0.0170 (5)	0.0130 (4)	−0.0004 (4)	0.0059 (4)	0.0011 (4)
C6	0.0168 (5)	0.0159 (5)	0.0131 (4)	−0.0014 (4)	0.0053 (4)	−0.0014 (3)
C7	0.0144 (4)	0.0150 (5)	0.0148 (4)	0.0015 (4)	0.0071 (4)	0.0006 (3)
C8	0.0190 (5)	0.0183 (5)	0.0149 (4)	−0.0017 (4)	0.0067 (4)	0.0015 (4)
C9	0.0174 (5)	0.0171 (5)	0.0134 (4)	−0.0021 (4)	0.0037 (4)	0.0007 (4)
C10	0.0160 (5)	0.0170 (5)	0.0170 (4)	0.0008 (4)	0.0055 (4)	0.0001 (4)
C11	0.0138 (4)	0.0193 (5)	0.0168 (4)	−0.0004 (4)	0.0057 (4)	0.0007 (4)
C12	0.0164 (5)	0.0155 (5)	0.0176 (4)	−0.0020 (4)	0.0060 (4)	0.0003 (4)
C13	0.0220 (5)	0.0177 (5)	0.0203 (5)	−0.0009 (4)	0.0109 (4)	−0.0020 (4)
C14	0.0229 (5)	0.0196 (5)	0.0186 (5)	−0.0038 (4)	0.0117 (4)	−0.0009 (4)
C15	0.0252 (5)	0.0206 (5)	0.0209 (5)	−0.0015 (4)	0.0096 (4)	0.0056 (4)
C16	0.0274 (6)	0.0153 (5)	0.0298 (6)	−0.0013 (4)	0.0158 (5)	−0.0019 (4)

### Geometric parameters (Å, °)

**Table:**

O1—C7	1.2310 (13)	C5—H5A	0.9300
O2—C4	1.3550 (12)	C6—H6A	0.9300
O2—C15	1.4246 (13)	C8—C9	1.4588 (15)
O3—C11	1.3656 (13)	C8—H8A	0.9300
O3—H1O3	0.87 (2)	C9—C14	1.3918 (15)
O4—C12	1.3631 (13)	C9—C10	1.4024 (15)
O4—C16	1.4300 (14)	C10—C11	1.3779 (15)
N1—C7	1.3523 (13)	C10—H10A	0.9300
N1—N2	1.3847 (12)	C11—C12	1.4046 (15)
N1—H1N1	0.887 (17)	C12—C13	1.3858 (15)
N2—C8	1.2808 (14)	C13—C14	1.3906 (15)
C1—C6	1.3930 (14)	C13—H13A	0.9300
C1—C2	1.3980 (14)	C14—H14A	0.9300
C1—C7	1.4877 (14)	C15—H15A	0.9600
C2—C3	1.3796 (15)	C15—H15B	0.9600
C2—H2A	0.9300	C15—H15C	0.9600
C3—C4	1.3978 (14)	C16—H16A	0.9600
C3—H3A	0.9300	C16—H16B	0.9600
C4—C5	1.3907 (14)	C16—H16C	0.9600
C5—C6	1.3888 (14)		
			
C4—O2—C15	117.11 (9)	C14—C9—C10	119.41 (10)
C11—O3—H1O3	107.6 (13)	C14—C9—C8	118.29 (10)
C12—O4—C16	115.78 (9)	C10—C9—C8	122.28 (10)
C7—N1—N2	119.91 (9)	C11—C10—C9	120.04 (10)
C7—N1—H1N1	121.5 (11)	C11—C10—H10A	120.0
N2—N1—H1N1	116.8 (11)	C9—C10—H10A	120.0
C8—N2—N1	113.42 (9)	O3—C11—C10	119.06 (10)
C6—C1—C2	118.77 (10)	O3—C11—C12	120.68 (10)
C6—C1—C7	123.40 (9)	C10—C11—C12	120.25 (10)
C2—C1—C7	117.83 (9)	O4—C12—C13	125.55 (10)
C3—C2—C1	120.90 (9)	O4—C12—C11	114.57 (9)
C3—C2—H2A	119.6	C13—C12—C11	119.88 (10)
C1—C2—H2A	119.6	C12—C13—C14	119.76 (10)
C2—C3—C4	119.85 (9)	C12—C13—H13A	120.1
C2—C3—H3A	120.1	C14—C13—H13A	120.1
C4—C3—H3A	120.1	C13—C14—C9	120.64 (10)
O2—C4—C5	124.53 (9)	C13—C14—H14A	119.7
O2—C4—C3	115.62 (9)	C9—C14—H14A	119.7
C5—C4—C3	119.85 (10)	O2—C15—H15A	109.5
C6—C5—C4	119.82 (9)	O2—C15—H15B	109.5
C6—C5—H5A	120.1	H15A—C15—H15B	109.5
C4—C5—H5A	120.1	O2—C15—H15C	109.5
C5—C6—C1	120.78 (9)	H15A—C15—H15C	109.5
C5—C6—H6A	119.6	H15B—C15—H15C	109.5
C1—C6—H6A	119.6	O4—C16—H16A	109.5
O1—C7—N1	123.74 (10)	O4—C16—H16B	109.5
O1—C7—C1	121.37 (9)	H16A—C16—H16B	109.5
N1—C7—C1	114.89 (9)	O4—C16—H16C	109.5
N2—C8—C9	122.15 (10)	H16A—C16—H16C	109.5
N2—C8—H8A	118.9	H16B—C16—H16C	109.5
C9—C8—H8A	118.9		
			
C7—N1—N2—C8	−154.92 (10)	N1—N2—C8—C9	179.20 (9)
C6—C1—C2—C3	0.60 (16)	N2—C8—C9—C14	−158.09 (11)
C7—C1—C2—C3	−178.84 (10)	N2—C8—C9—C10	20.44 (16)
C1—C2—C3—C4	0.97 (17)	C14—C9—C10—C11	0.05 (15)
C15—O2—C4—C5	−0.76 (16)	C8—C9—C10—C11	−178.47 (9)
C15—O2—C4—C3	179.62 (10)	C9—C10—C11—O3	−178.69 (9)
C2—C3—C4—O2	177.65 (10)	C9—C10—C11—C12	1.23 (15)
C2—C3—C4—C5	−2.00 (16)	C16—O4—C12—C13	10.02 (15)
O2—C4—C5—C6	−178.16 (10)	C16—O4—C12—C11	−170.42 (9)
C3—C4—C5—C6	1.45 (16)	O3—C11—C12—O4	−1.02 (14)
C4—C5—C6—C1	0.14 (16)	C10—C11—C12—O4	179.07 (9)
C2—C1—C6—C5	−1.16 (16)	O3—C11—C12—C13	178.58 (10)
C7—C1—C6—C5	178.25 (10)	C10—C11—C12—C13	−1.34 (16)
N2—N1—C7—O1	−0.80 (16)	O4—C12—C13—C14	179.72 (10)
N2—N1—C7—C1	178.78 (9)	C11—C12—C13—C14	0.17 (16)
C6—C1—C7—O1	−155.95 (10)	C12—C13—C14—C9	1.11 (16)
C2—C1—C7—O1	23.46 (15)	C10—C9—C14—C13	−1.22 (16)
C6—C1—C7—N1	24.46 (14)	C8—C9—C14—C13	177.35 (10)
C2—C1—C7—N1	−156.13 (10)		

### Hydrogen-bond geometry (Å, °)

**Table:**

*Cg*1 is the centroid of the C1–C6 ring.

**Table:**

*D*—H···*A*	*D*—H	H···*A*	*D*···*A*	*D*—H···*A*
O3—H1O3···O4	0.87 (2)	2.18 (2)	2.6692 (13)	115.9 (18)
N1—H1N1···O1^i^	0.887 (18)	1.992 (18)	2.8698 (13)	170.0 (16)
C8—H8A···O1^i^	0.93	2.47	3.2780 (15)	145
C15—H15C···Cg1^ii^	0.96	2.72	3.5664 (15)	148
C16—H16B···Cg1^iii^	0.96	2.76	3.4366 (16)	128

Symmetry codes:  (i) *x*, −*y*+3/2, *z*−1/2; (ii) −*x*+1, −*y*+2, −*z*; (iii) *x*, *y*−1, *z*.
